# Cranial defect and pneumocephalus are associated with significant postneurosurgical positional brain shift: evaluation using upright computed tomography

**DOI:** 10.1038/s41598-022-13276-0

**Published:** 2022-06-21

**Authors:** Keisuke Yoshida, Masahiro Toda, Yoshitake Yamada, Minoru Yamada, Yoichi Yokoyama, Kei Tsutsumi, Hirokazu Fujiwara, Kenzo Kosugi, Masahiro Jinzaki

**Affiliations:** 1 Department of Neurosurgery, Keio University School of Medicine, Tokyo, Japan; 2 Department of Radiology, Keio University School of Medicine, Tokyo, Japan; 3 Department of Neurosurgery, Mihara Memorial Hospital, Gunma, Japan

**Keywords:** Brain injuries, Neurological disorders

## Abstract

Only few studies have assessed brain shift caused by positional change. This study aimed to identify factors correlated with a large postneurosurgical positional brain shift (PBS). Sixty-seven patients who underwent neurosurgical procedures had upright computed tomography (CT) scan using settings similar to those of conventional supine CT. The presence of a clinically significant PBS, defined as a brain shift of ≥ 5 mm caused by positional change, was evaluated. The clinical and radiological findings were investigated to identify factors associated with a larger PBS. As a result, twenty-one patients had a clinically significant PBS. The univariate analysis showed that supratentorial lesion location, intra-axial lesion type, craniectomy procedure, and residual intracranial air were the predictors of PBS. Based on the multivariate analysis, craniectomy procedure (*p* < 0.001) and residual intracranial air volume (*p* = 0.004) were the predictors of PBS. In a sub-analysis of post-craniectomy patients, PBS was larger in patients with supratentorial craniectomy site and parenchymal brain injury. A large craniectomy area and long interval from craniectomy were correlated with the extent of PBS. In conclusion, patients who undergo craniectomy and those with residual intracranial air can present with a large PBS. In post-craniectomy patients, the predisposing factors of a large PBS are supratentorial craniectomy, presence of parenchymal injury, large skull defect area, and long interval from craniectomy. These findings can contribute to safe mobilization among postneurosurgical patients and the risk assessment of sinking skin flap syndrome.

## Introduction

Thus far, posture-induced intracranial changes have not been comprehensively evaluated. The recent development in upright computed tomography (CT) scan has helped in the objective evaluation of posture-related changes in the body with high-quality images comparable to those of a conventional supine CT scan^1^. In previous studies, the intracranial structures of normal volunteers slightly shifted caudally and posture-induced changes could be significant in patients with conditions such as sinking skin flap syndrome (SSFS) or supracerebellar arachnoid cyst^2–4^. However, there are no substantial studies regarding positional brain shift (PBS) in postneurosurgical patients. During neurosurgical procedures, we disconnect the structures fixating the brain to the surrounding structures by cutting the cranium, detaching dural adhesion, dissecting arachnoids, draining the cerebrospinal fluid (CSF), and even resecting parts of the brain parenchyma. Whether these surgical methods affect the stability of intracranial structures has not been assessed yet. If significant PBSs can occur in certain conditions, we must focus on the possible deteriorating effects of postural changes. The current study aimed to evaluate posture-related intracranial changes and factors that may be correlated with a significant PBS in postneurosurgical patients. We hypothesized that invasive intraoperative procedures requiring a larger craniotomy or craniectomy can lead to a greater postneurosurgical PBS.

## Methods

This prospective single-arm exploratory interventional study was approved by the Keio University School of Medicine Ethics Committee (Approval Number: 20180036). The research was performed in accordance with the Declaration of Helsinki for investigations involving human subjects. A written informed consent was obtained from all participants.

### Patient population

Patients aged 20 years or older who underwent neurosurgical procedures at our institution were prospectively included if they can undergo upright CT scan with consideration of their schedules and clinical conditions. All patients understood the purpose of the study, and the exclusion criteria were as follows: confirmed or suspected pregnancy and inability to maintain a stable sitting position.

### Image acquisition

The patients underwent upright CT scan (prototype TSX-401R; Canon Medical Systems, Otawara, Japan)^1,2,5–7^ in sitting position and supine CT scan (Aquilion ONE; Canon Medical Systems) in supine position in prospectively randomized orders. Upright CT scan has 320 detector rows, and its performance is comparable to that of conventional supine CT scan, as described previously^1^. Patients were instructed not to move during the scans after an unforced inhalation. Both CT scans were performed at 120 kVp, with a noise index of 4 for 5 mm slice thickness. CT scan images with a cross-sectional thickness of 0.5 mm and cross-section of 0.5 mm were obtained. Image reconstruction was performed using Adaptive Iterative Dose Reduction 3D (Canon Medical Systems), which reduced the radiation dose^8^.

All patients underwent upright CT scan using settings similar to those of conventional supine CT scan performed for clinical purposes. Since our department conducts CT scan on postoperative days 6 or 7, as a routine test before discharge, upright and supine CT scans were performed simultaneously during that time. As an exception, in patients with craniectomy, a set of CT scans was conducted at various timings as they were scheduled on a case-by-case basis according to the timing of cranioplasty.

### Radiological assessment

**Figure 1:**
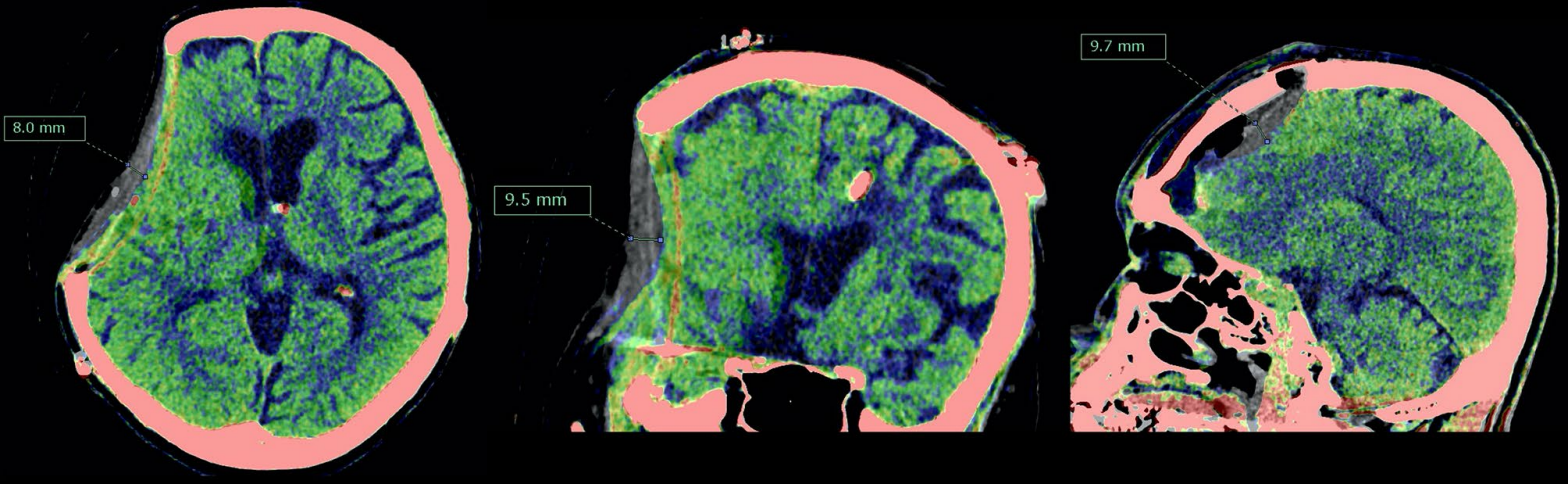
Positional brain shift measurement. Supine (black and white) and upright (mapped with color) images were compared after automatic rigid registration of two datasets based on cranial bones. This enabled three-dimensional assessment of positional brain shift.

The CT scan images were processed and analyzed using a three-dimensional image analyzing software (Synapse Vincent version 5.3; FUJIFILM Medical Co., Ltd., Lexington, MA, the USA). In a previous study, PBS in normal volunteers was 2.10 ± 0.86 mm^2^. In addition, other previous studies have used 5 mm as a cutoff value in the evaluation of brain shift^9–11^. Thus, in this study, a clinically significant PBS was defined as a brain shift of ≥ 5 mm in any region caused by positional change from supine to upright. Radiological assessment was performed by two experienced board-certified neurosurgeons, who compared supine and upright images after automatic rigid registration of two datasets based on cranial bones (Fig. 1)^2^. PBS was evaluated using multiplanar reconstruction of three-dimensional volume data. Result discrepancies were resolved via discussions to ensure a consensus decision. In a sub-analysis of patients with craniectomy, the maximal PBS was assessed in the cortical surface with the largest PBS within the skull defect area.

Using a software, the total intracranial volume was semiautomatically calculated as volume in the cranium above the basion-opisthion line. Intracranial CSF volume was calculated by segmenting the intracranial structure using a threshold of 0–15 Hounsfield units^12,13^. Relative intracranial CSF volume was calculated by dividing the estimated intracranial CSF volume by the intracranial volume^14^. The craniectomy area was evaluated via manual measurements using a software.

### Statistical analysis

All statistical analyses were performed using the SPSS software version 26 (IBM SPSS Statistics; IBM Corp., Armonk, NY, the USA). Continuous variables were expressed as mean ± standard deviation or median (interquartile range) and were analyzed using the Mann–Whitney *U* test. Two-tailed Fisher’s exact test was used for categorical variables. Variables with a *p* value of < 0.10 in the univariate analyses were used in the multivariate analysis. Multivariate logistic regression models were established using a forward stepwise selection with the likelihood-ratio criterion, where the final model contained only significant variables (*p* < 0.05). Data in the multivariate logistic regression analysis were presented as odds ratios (OR) and their respective 95% confidence intervals (CIs). Correlations between the numerical parameters were assessed using the Pearson’s correlation test. A *p*-value of < 0.05 was considered statistically significant.

## Results

In total, 67 patients (38 men and 29 women; mean age: 58.3 ± 14.9 years; mean body mass index: 23.4 ± 3.9 kg/m^2^) were included in the study. All participants well tolerated the supine and upright CT scans, and no adverse events were observed. No patients showed systemic hypotension before and after the scans.

The types of surgery were as follows: craniotomy, n = 43 (supratentorial, n = 35 and infratentorial, n = 8); craniectomy, n = 13 (supratentorial, n = 10 and infratentorial, n = 3); and less-invasive procedures, n = 11 (endoscopic endonasal, n = 8 and burr hole, n = 3). The underlying conditions were brain tumor in 43 (meningiomas, n = 20; gliomas, n = 9; pituitary tumors, n = 6; schwannomas, n = 4; and other types, n = 3), cerebrovascular disease in 6, trauma in 7, infection in 6, and other diseases in 5 patients. The baseline characteristics of patients are summarized in Table 1. Post-craniectomy patients (n = 13) underwent supine and upright CT scans a median of 12 days after operations (interquartile range: 7–136 days). Otherwise, four patients underwent CT scan on postoperative day 6, and all other patients on day 7.

**Table 1:** Baseline patient characteristics.

	n = 67
Age (mean ± SD)	58.3 ± 14.9
Sex (F/M)	29 / 38
Height, (mean ± SD, cm)	164.3 ± 8.9
Weight (mean ± SD, kg)	62.9 ± 10.6
Body mass index (mean ± SD)	23.4 ± 3.9
Post-craniotomy (%, supratentorial/infratentorial)	43 (64%, 35/8)
Post-craniectomy (%, supratentorial/infratentorial)	13 (19%, 10/3)
Post-less invasive surgery (burr hole/endoscopic endonasal)	11
Lesion types (tumor*/aneurysm/others)	43/6/14

*SD* standard deviation.

*Meningiomas, n = 20; gliomas, n = 9; pituitary tumors, n = 6; schwannomas, n = 4; and other types, n = 3.

**Figure 2:**
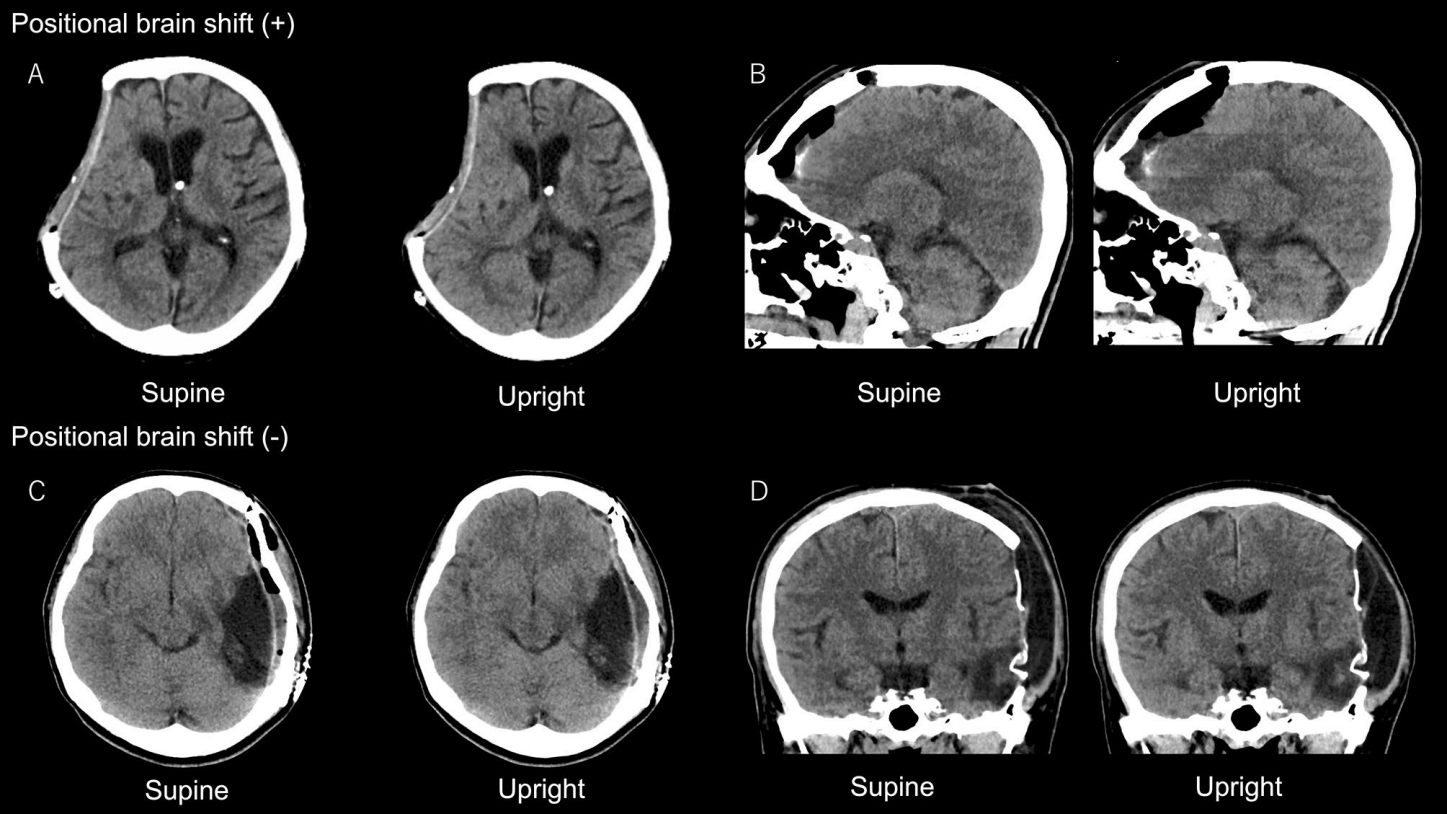
Examples of cases with (**A**, **B**) and without (**C**, **D**) positional brain shift (PBS). (**A**) A 70-year-old woman with ventriculoperitoneal shunt, who underwent craniectomy because of surgical site infection. Computed tomography (CT) scan images obtained on postoperative day (POD) 7 showed a remarkable PBS. (**B**) A 53-year-old man who underwent frontal craniotomy for clipping of a distal anterior cerebral artery aneurysm. In total, 14.9 mL of residual air, which moved upward according to positional change, thereby compressing the frontal lobe, was evident on CT scan images on POD 7. (**C**) A 41-year-old woman who underwent resection of left temporal glioma. CT scan images on POD 7 did not show PBS around the resection cavity. (**D**) A 55-year-old man who underwent craniectomy for traumatic brain injury. CT scan images on POD 17 did not show PBS. However, subcutaneous fluid collection indicated a positional shift.

In total, 21 patients had a clinically significant PBS (Fig. 2A,B). In 46 patients, PBS was not remarkable, and it measured < 5 mm (Fig. 2C,D). Whether demographic, clinical, and radiological variables can be predictors of PBS was analyzed (Table 2). A univariate analysis showed that supratentorial lesion location (*p* < 0.001), intra-axial lesion type (*p* = 0.002), craniectomy procedure (*p* = 0.017), and large intracranial air volume (7.23 ± 6.92 vs 0.51 ± 1.18 mL, *p* < 0.001) were significant predictors of PBS.

**Table 2:** Comparison between patients with and without positional brain shift (≥ 5 mm).

Variable	With PBS (n = 21)	Without PBS (n = 46)	*p* value univariate analysis	Multivariate analysis	
				OR (95% CI)	*p* value
**Patient characteristics**					
Age (year)	56.7 ± 16.0	59.5 ± 8.6	0.433		
Sex (n, F/M)	9/12	20/26	0.962		
Height (cm)	165.0 ± 9.2	163.1 ± 8.6	0.421		
Weight (kg)	59.6 ± 9.1	64.0 ± 11.3	0.234		
Body mass index (kg/m^2^)	21.9 ± 3.1	24.1 ± 4.2	0.089		
**Lesion characteristics**					
Lesion location (supratentorial/infratentorial)	21/0	28/18	** < 0.001**		
Lesion type (intra-axial/extra-axial)	9/12	4/42	**0.002**		
Lesion size (mm)	28.9 ± 20.8	27.2 ± 18.2	0.920		
**Operative variables**					
Craniotomy	12 (57%)	31 (67%)	0.426		
Craniectomy	8 (38%)	5 (11%)	**0.017**	**69.4 (7.7–623.3)**	** < 0.001**
Less-invasive surgery (endoscopic endonasal, burr hole)	1 (5%)	10 (22%)	0.152		
**Radiological variables in supine computed tomography**					
Total intracranial volume (L)	1.43 ± 0.15	1.43 ± 0.16	0.823		
Intracranial air volume (mL)	7.23 ± 6.92	0.51 ± 1.18	** < 0.001**	**2.0 (1.3–3.1)**	**0.004**
Relative intracranial cerebrospinal fluid volume (%)	8.9 ± 3.7	9.6 ± 4.1	0.756		

Significant values are in [bold].

*CI* confidence interval, *OR* odds ratio, *PBS* positional brain shift.

A multivariate analysis of body mass index, supratentorial lesion location, intra-axial lesion type, craniectomy procedure, and intracranial air volume was conducted. Based on a logistic regression analysis, craniectomy procedure (*p* < 0.001, OR = 69.4, 95% CI 7.7–623.3) and large residual intracranial air volume (*p* = 0.004, OR = 2.0, 95% CI 1.3–3.1) were the independent predictors of PBS.

In a sub-analysis of 13 post-craniectomy patients (Table 3), the mean maximal PBS in the craniectomy region was 7.8 ± 3.8 mm. Compared with infratentorial craniectomy, supratentorial craniectomy was significantly associated with a larger PBS (7.8 ± 3.8 vs 2.1 ± 0.6 mm, *p* = 0.018). Patients with parenchymal brain injury had a significantly larger PBS than those without (9.4 ± 4.1 vs 4.1 ± 2.3 mm, *p* = 0.022). In a correlation analysis, a large skull defect area (*r* = 0.700, *p* = 0.008) and long interval from craniectomy (*r* = 0.618, *p* = 0.024) were positively correlated with the extent of PBS. There were no statistically significant correlations between craniectomy side, age, body mass index, CSF diversion device implantation, and relative CSF volume. Moreover, 2 of 13 patients presented with orthostatic symptoms. One patient had objective symptom, particularly hemiparesis that worsened in upright position, and another experienced subjective postural symptom (dizziness). Although not statistically significant, symptomatic patients had a large PBS (11.5 ± 3.3 vs 5.6 ± 3.7 mm, *p* = 0.076).

**Table 3:** Sub-analysis of post-craniectomy patients.

Categorical variable	n = 13	Maximal positional brain shift	*p*
Sex (women/men)	5/8	4.3 ± 3.1/7.9 ± 4.3	0.284
Site (supratentorial/infratentorial)	10/3	7.8 ± 3.8/2.1 ± 0.6	**0.018**
Parenchymal brain injury (with/without)	6/7	9.4 ± 4.1/4.1 ± 2.3	**0.022**
CSF diversion device (with/without)	1/12	9.5 ± 0/6.3 ± 4.2	0.285
Postural symptom (with/without)	2/11	11.5 ± 3.3/5.6 ± 3.7	0.076

Continuous variable		Correlation coefficient	*p*
Age (year)	52.4 ± 16.9	0.265	0.382
Body mass index (kg/m^2^)	24.7 ± 4.4	− 0.417	0.157
Craniectomy area (mm^2^)	6296 ± 4811	0.700	**0.008**
Interval from craniectomy (days)	12 (7–136)	0.618	**0.024**
Relative intracranial CSF volume (%)	9.1 ± 4.6	0.404	0.171

Significant values are in [bold].

Data are presented as n, mean ± standard deviation, or median (interquartile range).

*CSF* cerebrospinal fluid.

**Figure 3:**
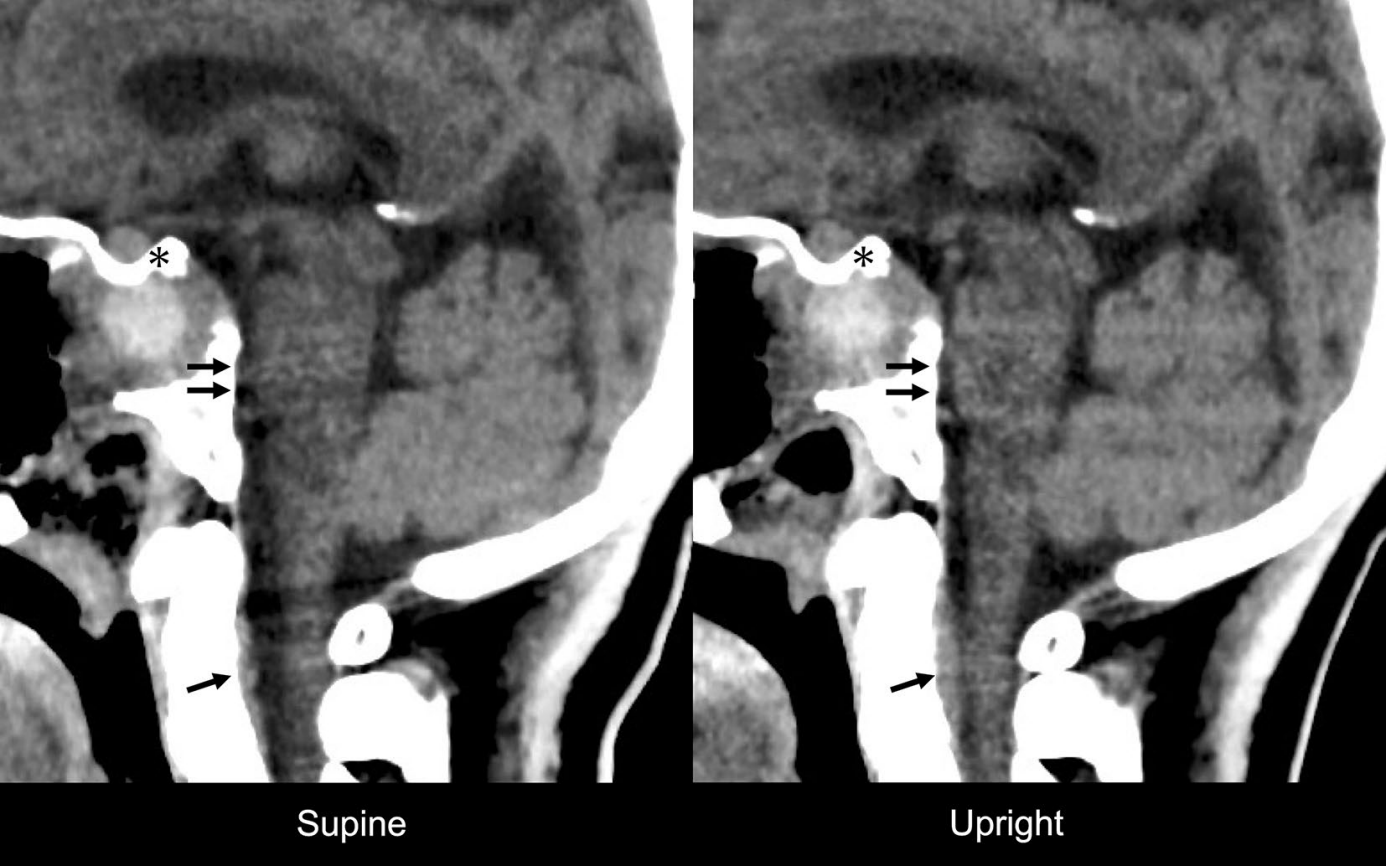
Computed tomography (CT) scan images with 0.5 mm slices of a 35-year-old woman who underwent cerebrospinal fluid leak closure using the endoscopic endonasal approach. Compared with supine imaging, upright CT scan revealed PBS to the ventrocaudal direction, distension of the anterior internal vertebral venous plexus (arrow), effacement of the prepontine cistern (double arrow), and enlargement of the pituitary gland (asterisk). See also Video 1.

One patient with Marfan syndrome who underwent endoscopic endonasal surgery for CSF leak closure from clival dural defect had suspected underlying intracranial hypotension with orthostatic headache preoperatively. Upright CT scan revealed a significant PBS to the ventrocaudal direction without pneumocephalus or cranial defect. Moreover, a remarkable distension of the anterior internal vertebral venous plexus was observed. Upright CT scan showed other typical signs of intracranial hypotension including enlargement of the pituitary gland, effacement of the prepontine cistern, and caudal brain shift (Fig. 3, Supplementary Video 1). However, these findings were not observed on supine CT scan.

## Discussion

The current study showed that craniectomy procedure and large residual air volume are significantly associated with intracranial shift caused by postural change in postneurosurgical patients. In addition, in post-craniectomy patients, supratentorial bone defect and presence of parenchymal brain injury were associated with PBS, and a large skull defect area and a long interval from craniectomy were correlated with a larger PBS.

Previous studies using conventional horizontal imaging have reported postural brain displacements in supine versus prone or lateral positions^15–17^, whereas until recently, only few studies have evaluated intracranial positional shifts in supine versus upright posture. Although early mobilization is recommended after surgeries, safety during mobilization has not been completely validated. Because most postneurosurgical patients undergo mobilization without complications, intraoperative procedures may not affect the brain stability to a clinically significant degree in most cases. However, after craniectomy or in cases involving residual intracranial air, PBS, which is not observed from the outside of the cranium, should be considered.

### PBS in post-craniectomy patients and its association with SSFS

The skull is supporting intracranial structures and is protecting them from not only mechanical damages but also posture-related physiological changes. Craniectomy partially removes this rigid barrier. Thus, the intracranial structures are more easily affected by the surrounding atmospheric pressure. The SSFS, or the syndrome of the trephined, is characterized by neurological disturbances in post-craniectomy patients that resolves after cranioplasty, and its pathophysiology is yet to be elucidated^18–24^. This condition is associated with a sunken skin flap in the skull defect region and is presumably caused by the distortion of underlying brain tissues^23^. Meanwhile, patients can be symptomatic even without radiological signs in supine position^14,19,25^. Aggravation of symptoms in upright position is a typical characteristic of SSFS^26,27^, and a previous case report has shown that upright imaging is effective in obtaining a diagnosis^3^. Although there was no statistically significant difference regarding the extent of PBS between patients with and without symptoms probably due to the small number of symptomatic individuals, it is reasonable to consider that a large PBS is responsible for orthostatic symptoms in SSFS.

This study revealed that large skull defect, long interval from craniectomy, and presence of parenchymal brain injury are associated with PBS. Previous studies have shown that these are also the risk factors of SSFS^19,21,28^. By contrast, other previously reported predisposing factors of SSFS, such as age^25,28^, sex^19,28^, and small relative intracranial CSF volume^14^ were not associated with PBS in this study. In the current study, PBS after infratentorial craniectomy was small. This supports the previous notion that infratentorial craniectomy does not require cranioplasty, and this is consistent with the fact that there is no reported case of SSFS after infratentorial craniectomy. Since only one patient had an implanted ventriculoperitoneal shunt, we could not assess the effect of CSF diversion devices, which can be a cause of SSFS based on previous reports^19,21^. In this patient, a relatively large PBS was observed despite a short interval from the craniectomy (7 days) and a small-sized bone defect, indicating the role of the CSF diversion device in increasing the risk of PBS (Fig. 2A).

SSFS is likely to develop when the resistance of skin flap tension and intracranial pressure have been affected by atmospheric pressure. Therefore, an increase in the elastance of the skull defect region, mainly composed of the skin flap, brain parenchyma, and CSF, can make the craniectomy area more vulnerable to internal brain shift. Factors correlated with a larger PBS contribute to a lower regional elastance. These include the removal of a large part of a rigid skull, tissue scarring with atrophy in the skin flap, which occurs if there is a long interval from craniectomy, injured brain with loss of parenchymal volume, and use of CSF diversion devices that contribute to intracranial hypotension.

### PBS in postoperative pneumocephalus

Residual intracranial air was significantly associated with a large PBS. We intuitively understand that air in the restricted space moves according to positional changes. The specific gravity of air is quite lower than that of CSF. Therefore, intracranial air moves upward in upright position. When there is a large volume of air in the cranium, it can be space-occupying, which is enough to compress the adjacent brain. By contrast, the bridging veins in the convexity area are fixed to the brain surface and dura mater^29^. When the brain surface is compressed by air, these bridging veins might be stretched and torn in severe cases. With an accelerated motion with positional change, the tension affecting surrounding structures may increase, causing hemorrhage in the veins often located in the frontoparietal parasagittal region^30–32^. One report has shown that a torn bridging vein caused spontaneous subdural hematoma in a patient with pneumocephalus^33^. A certain number of cases involve postoperative ischemic or hemorrhagic complications of unknown cause after uneventful surgeries. These complications are often described as a venous cause with unvalidated etiologies. We assume that an increase in PBS can cause such complications. In patients with a large volume of residual intracranial air, rapid postural changes should be prevented, and possible stretching of the frontoparietal bridging veins while in upright position, which is not expected from examinations performed in supine position, must be considered. A study of PBS evaluated via supine versus prone MRI performed a similar assessment of the risk of bridging vein damage caused by positional change^17^. Major cortical and bridging veins do not show positional shifts compared with brain parenchyma.

Moreover, a related mechanism in the so-called remote hemorrhage after neurosurgical surgeries has been discussed^34,35^. The presence of postoperative pneumocephalus indicates that air has substituted the space previously occupied by something intraoperatively removed, such as resected mass lesion and drained CSF. One of the most convincing mechanisms is tearing or transient occlusion of stretched bridging or subcortical veins associated with CSF overdrainage and mechanical brain shift^34–36^. This is supported by the fact that surgery performed in sitting position is a risk factor of remote hemorrhage^34^. We believe that even patients who underwent surgeries in supine position can be affected by the same mechanical shift postoperatively due to PBSs. Although we routinely substitute artificial CSF for intracranial air before dural closure, the air cannot be removed completely. In addition, such a replacement maneuver cannot be utilized in the endoscopic endonasal approach. After endoscopic endonasal skull base surgery with massive intraoperative CSF leakage with severe pneumocephalus, the risk of PBS must be considered. Delayed mobilization might be recommended until the volume of residual air is decreased with absorption.

### Upright imaging in intracranial hypotension

Previous studies have shown that pneumocephalus concomitant with CSF hypovolemia or intracranial hypotension is an important radiological finding. This condition can lead to severe post-craniotomy complications, referred to as sinking brain syndrome^37^ or brain sag after craniotomy^38^. Because upright images were not obtained immediately or earlier after surgery, the effect of postoperative intracranial hypotension could not be evaluated. However, one patient with Marfan syndrome who underwent CSF leak closure presented with remarkable radiological changes based on positions. Upright imaging revealed typical findings of intracranial hypotension^39–41^. Whether these results are solely caused by intracranial hypotension due to CSF leakage or are correlated with connective tissue weakness in Marfan syndrome has not been validated^42^. Both factors might have affected positional radiological changes since both entities are closely correlated^43–45^. A previous report of six patients with intracranial hypotension caused by CSF leak showed no positional changes. However, the study conducted 0.6 Tesla MRI with a low spatial resolution, and only few patients were included^46^. Therefore, further studies targeting patients with spontaneous intracranial hypotension or orthostatic headache must be conducted to validate whether upright imaging is effective for diagnosing this rare but underdiagnosed pathology.

### Limitations

The current study had several limitations. First, there was selection bias in terms of patient selection in this single-arm exploratory study, which was performed at a single institution. We could not enroll patients with severe brain damage who have restricted activities or who cannot provide informed consent for the study. Thus, baseline diseases comprised a relatively large proportion of extra-axial tumors. In addition, we excluded patients who cannot maintain a stable upright position, including those with severe orthostatic symptoms.

Second, the timing of CT scans varied as the procedures must be performed based on the limited availability of the upright CT scanner and the hospital’s clinical workflow should not be disturbed. Therefore, most patients underwent upright and routine supine CT scan on postoperative days 6 or 7. CT scan images obtained in the earlier postoperative stage must have shown more significant changes according to positions due to a large volume of residual air with less refilled CSF and undeveloped postsurgical tissue adhesion. However, earlier scans were not feasible owing to safety concerns. In addition, post-craniectomy patients underwent CT scans at different timings because procedures were clinically scheduled according to the timing of the cranioplasty. Nevertheless, variations in the intervals from the craniectomy, in turn, contributed to the sub-analysis that used this interval as an important variable.

## Conclusion

Patients who underwent craniectomy and those with residual intracranial air can present with a large PBS. In post-craniectomy patients, supratentorial craniectomy, presence of parenchymal injury, large skull defect area, and long interval from craniectomy can be a risk factor for significant PBS. These findings can contribute in performing a safe mobilization among postneurosurgical patients and in the risk assessment of SSFS.

## Supplementary Information

Supplementary Video legend.

Supplementary Video 1.

## Data Availability

The datasets generated during the current study are available from the corresponding author on reasonable request.
