# Supplementary material for: Cranial defect and pneumocephalus are associated with significant postneurosurgical positional brain shift: evaluation using upright computed tomography
Source: Sci Rep. 2022 Jun 21;12:10482. doi: 10.1038/s41598-022-13276-0 (PMC9213471; doi:10.1038/s41598-022-13276-0)
Supplement: Supplementary file 1 — Supplementary Video legend. [file 41598_2022_13276_MOESM1_ESM.docx]

Supplementary Video 1. Supine and upright computed tomography scan datasets with 0.5-mm slices of the patient described in Figure 2 were registered based on cranial bones, and they were further assessed to observe the positional changes.
